# Effect of Pore Size Distribution and Amination on Adsorption Capacities of Polymeric Adsorbents

**DOI:** 10.3390/molecules26175267

**Published:** 2021-08-30

**Authors:** Wei Liu, Yuxi Zhang, Shui Wang, Lisen Bai, Yanhui Deng, Jingzhong Tao

**Affiliations:** Jiangsu Key Laboratory of Environmental Engineering, Jiangsu Academy of Environmental Sciences, 176# Jiangdong Beilu Road, Nanjing 210036, China; zhangyuxiNJU@163.com (Y.Z.); ws@vip.sina.com (S.W.); bailisen23@163.com (L.B.); jsshkylw@163.com (Y.D.); jshbtao@126.com (J.T.)

**Keywords:** resin, adsorption, pore size distribution, chemical modification, water treatment

## Abstract

Polymeric adsorbents with different properties were synthesized via suspension polymerization. Equilibrium and kinetics experiments were then performed to verify the adsorption capacities of the resins for molecules of various sizes. The adsorption of small molecules reached equilibrium more quickly than the adsorption of large molecules. Furthermore, the resins with small pores are easy to lower their adsorption capacities for large molecules because of the pore blockage effect. After amination, the specific surface areas of the resins decreased. The average pore diameter decreased when the resin was modified with either primary or tertiary amines, but the pore diameter increased when the resin was modified with secondary amines. The phenol adsorption capacities of the amine-modified resins were reduced because of the decreased specific area. The amine-modified resins could more efficiently adsorb reactive brilliant blue 4 owing to the presence of polar functional groups.

## 1. Introduction

Nowadays, large amounts of wastewater containing pollutants such as pesticides, dyes, heavy metals, and pharmaceuticals have been produced because of increased agricultural applications and the growth of the pharmaceutical and chemical industries [1]. Effluent containing even low concentrations of organic contaminants can be highly discernible and recalcitrant [2]. The large-scale production and widespread use of chemical substances can cause serious environmental problems, making it an important public concern [3]. Thus, the effective removal of organic pollutants from water sources is necessary for environmental security and public health.

Adsorption is a low-energy solid phase extraction technique that has been widely applied in industry in recent years. Various materials have been used as adsorbents, including mesoporous silica, natural zeolites and activated carbons [4,5]. However, these materials have some disadvantages that limit their application, such as unfavorable selective adsorption or poor regeneration [6].

Since the 1970s, functional polymers that have adsorption and separation capability have rapidly developed, and adsorbent resins have been widely used in various fields. Resins can adsorb organic matter through non-covalent bonding between molecules. The adsorbed molecules can then be eluted, thereby regenerating the resin to achieve the enrichment, separation and recovery of organic matter in wastewater [7,8,9]. For example, Zhu Shiyun et al. [10] used the strong anionic resin Amberlite IRA 402-OH to remove acetaldehyde from petrochemical production wastewater. The acetaldehyde removal efficiency from wastewater was 86%.

Adsorbent resins have been widely used to remove in dyes chemicals (such as benzene and naphthalene), pharmaceutical intermediates, and other organic compounds from wastewater [11]. Although existing adsorption resins can efficiently remove micromolecules and low water-soluble organic matter from wastewater, they are not effective for the adsorption of large molecules or organic matter with complex molecular structures. Therefore, a series of resins with different pore size distributions and high-adsorption capacity chemical groups were synthesized to address this problem. The adsorption behavior and effects on adsorption properties were studied.

Adsorbent resins can be synthesized via addition polymerization and condensation polymerization. The design of the pore structure in the resin synthesis is important and includes the pore volume, pore size, pore distribution, and specific surface area [12]. Many factors can affect the pore structure. For instance, when synthesizing macroporous adsorbents, the polarity, pore size, pore distribution, and specific surface area of the resin can be adjusted by controlling the polymerization conditions, such as the crosslinking agent, amount and type of porogen, and monomer composition [13,14]. The pore structure plays an important role in the adsorption ability of resins.

In addition to the pore diameter, the chemical structure of the resin also has an important effect on its adsorption properties. Li et al. [15] carbonized XAD-4 resin and found that the modified resin had good adsorption of phenolic compounds. Huang et al. [16] synthesized a new diethylenetriamine-modified hyper-cross-linked polystyrene resin with improved adsorptive removal of phenol. Li et al. [17] synthesized anion-exchange resins with different trialkylammonium groups, which significantly improved the nitrate adsorption capacity and improved the humic acid anti-fouling performance. Thus, we modified synthesized resins with amines and increase the resins adsorption for stronger polar and bigger molecules.

## 2. Materials and Methods

### 2.1. Chemicals

Divinylbenzene (DVB, 83.5%) and divinylbenzene (DVB, 50.0%) was purchased from Shandong Dongda Chemicals Company (Shandong, China) which were used as monomers. Toluene (>99.7%, Shanghai Chemical Reagent Company), liquid wax (>99.5%, Shanghai Chemical Reagent Company), n-heptane (>99.7%, Shandong Dongda Chemicals Company) and hexadecanol (>99.7%, Shanghai Chemical Reagent Company) were used as porogen. Gelatin (Photographic grade) was purchased from Yancheng Dafeng Gelatin Company (Jiangsu, China). Benzoyl peroxide (>99.7%, Shanghai Chemical Reagent Company) was used as an initiator. Zinc chloride (>99.7%) as the catalyst for chloromethylation were purchased from Shanghai Chemical Reagent Company. Chloromethyl ether (>99.7%, Shanghai Chemical Reagent Company), chloroform (>99.7%, Nanjing Chemical Reagent Company) and methylal (>99.7%, Shanghai Chemical Reagent Company) were used as the swelling agent. Hexamethylenetetramine (>99.7%), methylamine aqueous solution (25%) and dimethylamine aqueous solution (33%) used for amino-modified were all purchased from Nanjing Chemical Reagent Company. HCl and NaOH were obtained from Shanghai Chemical Reagent Company. Phenol (>99.7%), 1-amino-4- bromoanthraquinone-2sulfonic acid (ABS acid, >99.7%), and reactive brilliant blue 4 (RBB4, >99.5%) used as adsorbates were also purchased from Nanjing Chemical Reagent Company. Their structure and molecular sizes of them were estimated by WinMopac7.21, and the results are shown in Figure S1.

### 2.2. Preparation

**Figure 1:**
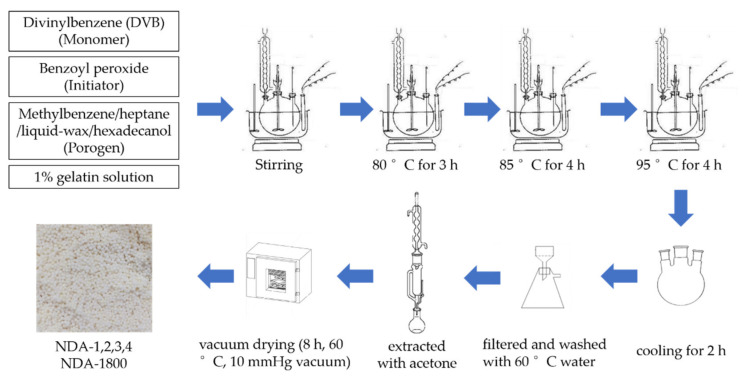
The flow diagram of resin synthesis.

An aqueous phase (200.0 g) solution consisting of 1% gelatin and an oil phase consisting of divinylbenzene (DVB, 83.5% or 50.0%), benzoyl peroxide (initiator) and different porogen were added to a 500-mL flask. The stirring speed was 300 rpm. The temperature was gradually increased to 80 °C for 3 h, then to 85 °C for 4 h, and finally to 95 °C for 4 h. After cooling for 2 h, the polymer beads were filtered with glass sand core vacuum filtration apparatus (50 mesh) and washed with hot water (60 °C). The obtained resins were NDA-1, NDA-2, NDA-3, NDA-4 and NDA-1800 for different monomer concentrations, porogen proportion and monomer: porogen ratio. All the polymer beads were then extracted with acetone in a Soxhlet extractor for 8 h, following by vacuum drying for 8 h at 60 °C under a 10 mmHg vacuum, respectively. The reaction process, final product and corresponding reagents are shown in Figure 1 and Table S1.

Then, the primary aminated resin (NDA-1801), the secondary aminated resin (NDA-1802) and the tertiary aminated resin (NDA-1803) were prepared, respectively. NDA-1800 resin (300.0 g) was chloromethylated with chloromethyl ether (1800.0 g) by using zinc chloride (180.0 g) as the catalyst. For NDA-1801 preparation, the chloromethylated NDA-1800 resin (80.0 g) was then emerged in chloroform (120 mL) for 2 h, and then hexamethylenetetramine aqueous solution (50.0%, 331.7 g) was added. The pH value was adjusted to 12.0 with NaOH (10 mol/L). For the preparation of NDA-1802, NDA-1800 resin (80.0 g) was swelled in methylal (120 g) 2 h, and then the methylamine aqueous solution (25%) was added. The pH was adjusted to 12 with NaOH (10 mol/L). The solution was then incubated at 45 °C for 10 h. For the preparation of NDA-1803, NDA-1800 resin (80.0 g) was also swelled in methylal (120 g) 2 h. Then, the dimethylamine aqueous solution (33%) was added, and the pH was adjusted to 12 with NaOH (40%). The solution was incubated at 45 °C for 10 h. After the amination, all the obtained beads were washed with deionized water until the pH was neutral.

### 2.3. Characterization

The BET surface area (S_BET_) of the resin was calculated using the isothermal adsorption and desorption experiments of N_2_ at 77 K. The micropore volume (V_micro_) and the micropore area (S_micro_) were determined by the t-plot method [18]. When the relative pressure is close to 1 (0.98 in this experiment), the N_2_ adsorption amount (V_t_) corresponds to the total adsorption amount of micropores and mesopores in the resin. The mesopore volume (V_meso_) of the resin can be obtained by subtracting V_micro_ from the adsorption amount at this time. The average pore diameter in the mesoporous region was obtained through a BJH desorption experiment. The whole process was completed automatically by an accelerated surface area and porosimeter system (ASAP 2010, Micromeritics, Georgia USA). The exchange capacity was determined by acid-base titration. The pretreated resin was soaked in the HCl aqueous solution, and the capacity was obtained by NaOH titration of the residual HCl in the solution with phenolphthalein [19]. Fourier transform infrared (FTIR) spectroscopy was performed with an FTIR spectrophotometer (Nicolet 170 SX, Georgia USA) via KBr pressed-disk technique.

### 2.4. Adsorption Kinetics

A 0.1000 g aliquot of resin was accurately weighed into a 250-mL conical flask, and 100 mL of preheated (303 K) 500 mg·L^−1^ aqueous solution of adsorbate was added. The flask was sealed and placed on a constant temperature shaker set at 303 K and shaken at 130 rpm. Samples were removed at regular intervals to measure the concentration of the adsorbates via a high-performance liquid chromatography (Waters 600) and a UV-vis spectrophotometer (GBC UV/VIS 916).

### 2.5. Static Equilibrium Adsorption

A 0.1000 g aliquot of resin was weighed into a 250-mL conical flask. Different concentrations of adsorbate solution (100 mL) were added, and the mixtures were shaken for 48 h on a constant temperature shaker at 303 K and 130 rpm. The solute concentrations in the equilibrated solutions were then analyzed.

### 2.6. Analysis

Phenol was determined by a high-performance liquid chromatography (Waters 600). ABS acid and RBB4 were determined by a UV-vis spectrophotometer (GBC UV/VIS 916) at 486 nm and 605 nm, respectively.

The equilibrium adsorption capacity (*Q_e_* (mmol·g^−1^)) was calculated as follows:

(1) $$Q_{e}=\frac{V_{1}\left(C_{0}-C_{e}\right)}{MW}$$

where, *V_1_* (L) is the volume of the solution, *W* (g) is the mass of the dry resin, and *M* is the molar mass of the adsorbate molecule.

## 3. Results and Discussion

### 3.1. Characterization

The specific surface area and pore structure of the synthesized resins (NDA-1, NDA-2, NDA-3, and NDA-4) were measured. Their specific surface areas ranged from 448.0 to 565.1 m^2^·g^−1^.

**Figure 2:**
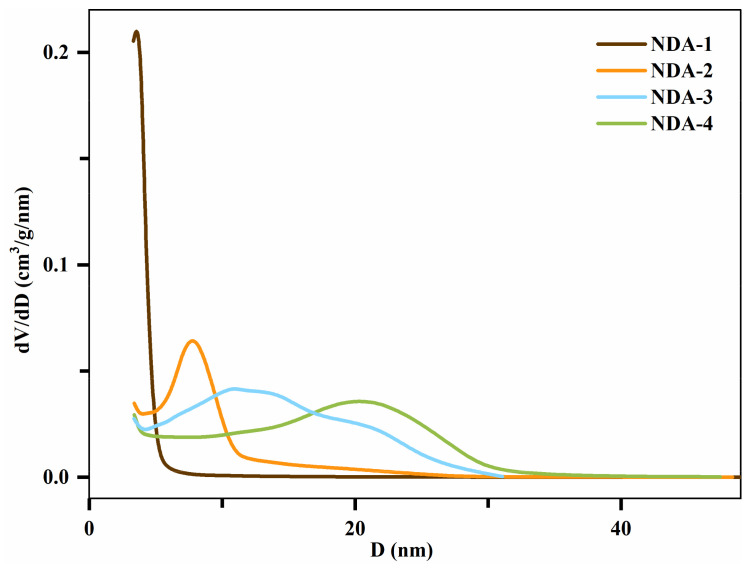
Mesopore diameter distribution of NDA-1, NDA-2, NDA-3, and NDA-4.

The characterization results of the resins are shown in Table 1. The mesopore diameter distribution was calculated using the Barret-Joyner-Halenda (BJH) method, as shown in Figure 2. NDA-2 had the largest specific surface area, and the other three resins had similar specific surface areas. The pore diameter distributions of NDA-1 and NDA-2 were relatively narrow, but those of NDA-3 and NDA-4 were relatively wide.

**Figure 3:**
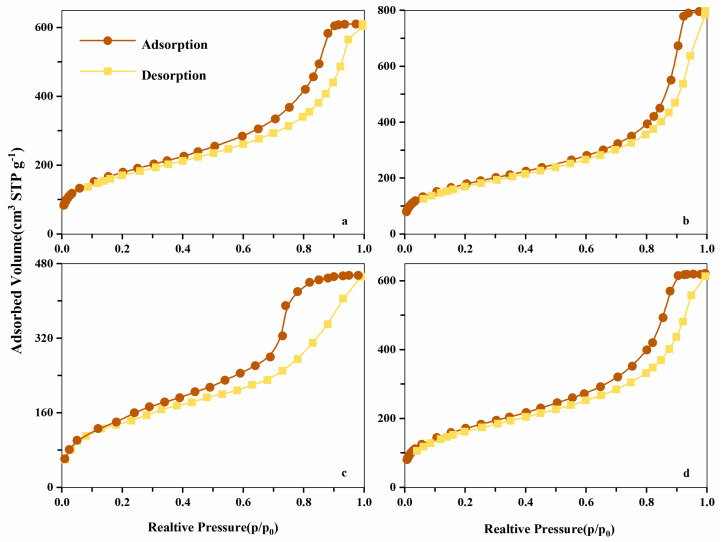
N_2_ adsorption isotherms of NDA-1 (**a**), NDA-2 (**b**), NDA-3 (**c**) and NDA-4 (**d**) resins at 77 K.

The N_2_ adsorption isotherms of the synthetic resins were mostly II and IV hybrid adsorption isotherms (see Figure 3). Micropore filling occurred when relative pressure (p/p_0_) was between 0.0 to 0.2 and the adsorption was low. When the relative pressure was slightly higher, the hysteresis curve appeared between the adsorption and desorption curves owing to the occurrence of multilayer adsorption and capillary condensation, indicating that most pores in the resins were mesopores [20]. The low range isotherms (at the logarithmic scale (p/p_0_) scale) were also presented in supporting information (Figure S2).

After modification of NDA-1800 with amines, their characterizations were determined. The infrared spectra of NDA-1800, NDA-1801, NDA-1802, and NDA-1803 resins are shown in Figure S3. Compared with the NDA-1800 resin, the resins modified by primary amines, secondary amines and tertiary amines had a very small peak around 672 cm^−1^; this was attributed to the chloromethyl peak. The peaks appearing near 1040 cm^−1^ and 1118 cm^−1^ were designated as C-N stretching vibration peaks, and they were not present for NDA-1800. In the modified resins, the peaks around 3445 cm^−1^ were N-H stretching vibration peaks, which indicated that amine groups had been successfully introduced to the three modified resins. Moreover, the chloride content of the resin after amination was decreased, indicating that the chlorine had been partly replaced by the amino groups (Table 2). The exchange capacities of the three modified resins were 1.3, 1.49, and 5.4 mmol·g^−1^ for NDA-1801, NDA-1802, and NDA-1803, effectively, showing that the number of exchangeable amino groups increased on the resin skeleton after reaction. Therefore, when contacted by adsorbate, the resins could provide exchange sites and electrostatic adsorption sites in addition to the π-π interactions of the resin skeleton.

**Figure 4:**
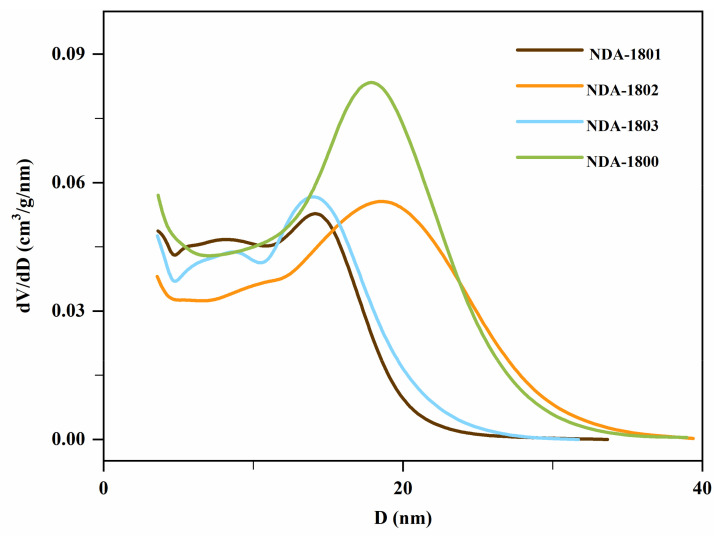
Mesopore diameter distribution of NDA-1800, NDA-1801, NDA-1802, and NDA-1803.

The data in Table 2 show that compared with NDA-1800, the specific surface areas of the resins modified with primary amines, secondary amines and tertiary amines were 26.0%, 26.3%, and 29.3% smaller, respectively. This was because during chloromethylation some part of the resin pores collapsed owing to the swelling action of chloromethyl ether, resulting in a decreased specific surface area. Figure 4 shows that the pore diameter of the resins also changed after modification with amine groups. The concentration pore diameters of resins modified with primary amines and tertiary amines slightly decreased to approximately 14 nm, while that of the resin modified with secondary amines slightly increased.

### 3.2. Adsorption Kinetics

The adsorption kinetic data were analyzed using pseudo-first-order and pseudo-second-order rate equations via nonlinear fitting [21,22]; Table 3 lists the corresponding parameters. The correlation coefficient (R^2^) suggested that the pseudo-second-order rate equation was a better fitting model.

**Figure 5:**
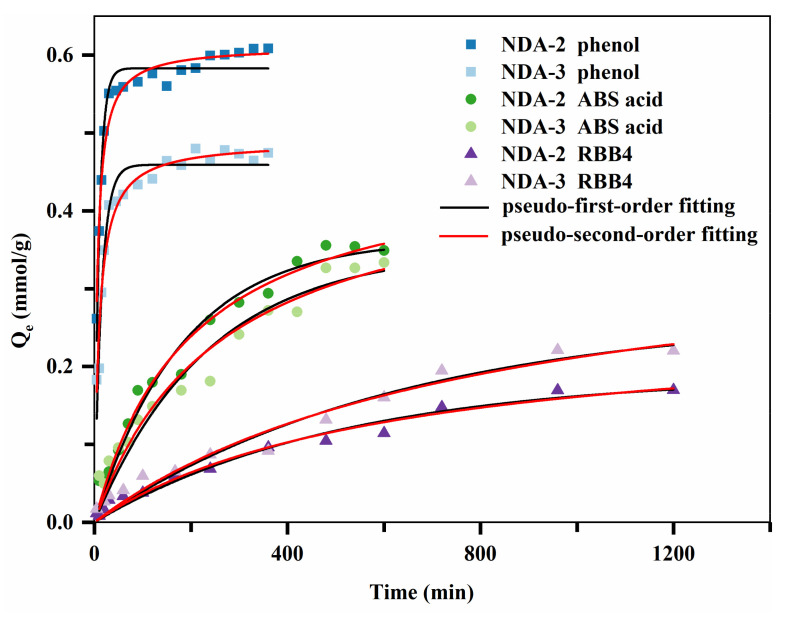
Adsorption kinetic curves of the three adsorbates.

Figure 5 shows the adsorption kinetic curves of the three adsorbates on NDA-2 and NDA-3. Phenol rapidly reached equilibrium in approximately 0.5 h, ABS acid reached equilibrium in approximately 8 h, and RBB4 reached equilibrium in approximately 20 h. The kinetics of the different adsorbates varied because the adsorption process on the inner surface of the resin is often very fast, and is mainly controlled by internal diffusion into the resin pores [23]. Owing to the relative size of the resin pores and the molecular size of phenol, the diffusion of phenol at the resin pore interface is similar to molecular diffusion in which there is free diffusion and a relatively high diffusion rate. However, RBB4 has a larger molecular volume, which leads to more frequent collisions with the pore wall, greater diffusion resistance, and a slower diffusion rate.

The rate constant, k_2_, of phenol adsorption was in the following order: NDA-2 > NDA-3, while this was the opposite for RBB4. The equilibrium adsorption capacities of NDA-2 for phenol and ABS acid were greater than those of NDA-3, while this was the opposite for RBB4. The pore diameter of NDA-2 was smaller than that of NDA-3, leading to a higher equilibrium adsorption capacity for micromolecules such as phenol and ABS acid. In contrast, NDA-3 had higher equilibrium adsorption capacity for the macromolecule RBB4. Therefore, the pore diameter distribution of the resin not only affected the adsorption rate, but also influenced the adsorption capacity of the resin for an adsorbate.

### 3.3. Static Adsorption Equilibrium

**Figure 6:**
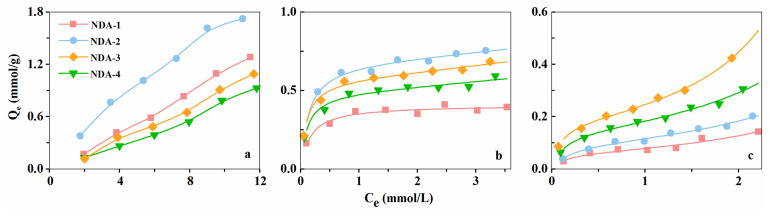
(**a**) Adsorption isotherms of phenol; (**b**) Adsorption isotherms of ABS acid; (**c**) Adsorption isotherms of RBB4.

We used a three-parameter multilayer adsorption model to describe the adsorption data [24]. The equation was as follows:

(2) $$Q_{e}=\frac{Q_{m}K_{1}C_{e}}{\left(1-K_{2}C_{e}\right)\left[1+\left(K_{1}-K_{2}\right)C_{e}\right]}$$

where *C_e_* (mmol·L^−1^) is the equilibrium concentration of adsorbate, *Q_e_* (mmol·g^−1^) is the adsorption capacity of resin, *Q_m_* (mmol·g^−1^) is the maximum first-layer adsorption capacity of resin, *K_1_* (L·mmol^−1^) is the first-layer adsorption equilibrium constant, and *K_2_* (L·mmol^−1^) is the multilayer adsorption equilibrium constant. The three-parameter multilayer adsorption model can be used to describe almost all S-type adsorption isotherms, including the BET isotherm and the Freundlich isotherm, which are typical for gas adsorption and organic matter adsorption. The equilibrium adsorption isotherms are shown in Figure 6, and Table 4 summarizes the corresponding parameters. The adsorption isotherms of phenol were approximately straight lines in the concentration range of our study and could not be simulated by this model.

Figure 6a shows the adsorption isotherms of phenol on the four resins. The order of adsorption capacity was NDA-2 > NDA-1 > NDA-3 > NDA-4. The NDA-2 resin had the highest adsorption capacity because it had the largest specific surface area. Although NDA-1, NDA-3 and NDA-4 had similar specific surface areas, the pore diameter distribution of NDA-1 was mainly concentrated in the small mesopore region. This resin is suitable for the adsorption of micromolecules such as phenol. However, the opposite was true for NDA-4, which had lowest adsorption capacity for phenol.

Figure 6b shows the adsorption isotherm of ABS acid on the four resins. The order of adsorption capacity was NDA-2 > NDA-3 > NDA-4 > NDA-1. Different from phenol, the adsorption capacity of NDA-1 for ABS acid was much lower than that of the other three resins. This was because the adsorption of ABS acid to NDA-1 was affected by pore hindrance, making part of the pore volume unusable. One reason for this phenomenon is that the pore size of NDA-1 was smaller than that of the adsorbate molecule; thus, the molecule cannot be adsorbed within the resin pores. Second, in the process of diffusion from the surface to the inner channels of the resin, the adsorbate molecules are partly adsorbed onto the pore walls. This can cause the channels become blocked, preventing the adsorbate from passing through the channels. As can be seen from the pore diameter distribution diagram, the pore diameter of NDA-1 was mainly distributed around approximately 3 nm, this is 2–3 times larger than the diameter of ABS acid. Therefore, the adsorption of this molecule caused pore obstruction.

Figure 6c shows the adsorption isotherm of RBB4 on the four resins. The order of adsorption capacity was NDA-3 > NDA-4 > NDA-2 > NDA-1. Although NDA-2 had the largest specific surface area, its adsorption capacity was far lower than that of NDA-3 and NDA-4. This was because the NDA-2 resin was also affected by pore hindrance. This effect was enhanced by the molecular aggregation of RBB4 [25]. Walker et al. [26] showed that the degree to which some acid dyes aggregate on the surface of activated carbon and bone charcoal could be greater than 10 molecules. Accordingly, it is understandable that the macromolecule RBB4 would experience pore hindrance on, NDA-1 and NDA-2 owing to small pore diameters of these resins.

As shown in Figure 6, although NDA-4 and NDA-3 had similar specific surface areas, pore volumes and average pore diameters, the adsorption capacity of NDA-4 was lower than that of NDA-3 for the three adsorbates. This was because the pore diameter of NDA-4 was larger than that of the adsorbates. Thus, pore size that is either too small or too large is not conducive to improving the adsorption capacity of a resin, and there was an optimal adsorption pore size for specific adsorbates.

The size distribution of the pores greatly influences the adsorption capacity of a resin. Only when the resin has a proper pore diameter can the adsorbate achieve good diffusion into the channels and be absorbed effectively. If the pore diameter is too large and the specific surface area is small, the resin will have a low adsorption capacity. If the pore diameter is too small, it will limit the diffusion of adsorbates and solvents, which enhances the shielding effect for molecules with larger diameters [27]. When the pore diameter of a micropore is less than several times the diameter of the adsorbate molecule, a potential shielding effect can occur. This can increase the interaction between the solid surface and gas molecules, thus enhancing adsorption [28]. Macropores, mesopores and micropores are typically categorized by pore diameters greater than 50 nm, 2–50 nm, and less than 2 nm, respectively. The surface of macropores contributes little to adsorption and only provides a channel for the diffusion of adsorbates and solvent. Mesopores can adsorb macromolecules and help micromolecules pass through the micropores.

Adsorption is related to the chemical structure of resin, regardless of the adsorption method. Regarding the adsorption mechanism, non-polar adsorbent resins adsorb molecules through van der Waals forces. By contrast, adsorption typically occurs via dipole–dipole and electrostatic interactions (including hydrogen bond and donor–acceptor interactions) for medium and strongly polar resins.

### 3.4. Adsorption Properties of the Modified Resins

**Figure 7:**
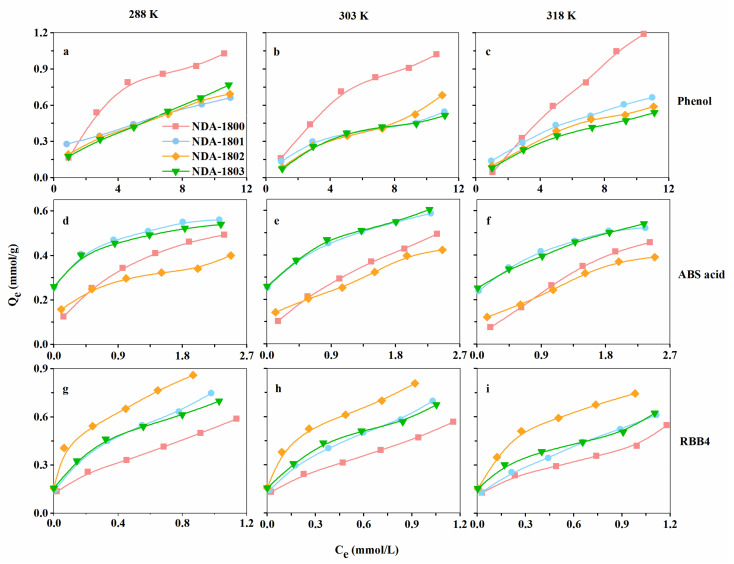
(**a**) Adsorption isotherms of phenol at 288 K; (**b**) Adsorption isotherms of phenol at 303 K; (**c**) Adsorption isotherms of phenol at 318 K; (**d**) Adsorption isotherms of ABS acid at 288 K; (**e**) Adsorption isotherms of ABS acid at 303 K; (**f**) Adsorption isotherms of ABS acid at 318 K; (**g**) Adsorption isotherms of RBB4 at 288 K; (**h**) Adsorption isotherms of RBB4 at 303 K; (**i**) Adsorption isotherms of RBB4 at 318 K.

The adsorption properties of the three amino-modified resins (NDA-1801, NDA-1802, and NDA-1803) for phenol, ABS acid and RBB4 were investigated at 288 K, 303 K, and 318 K and were compared with NDA-1800. The results are presented in Figure 7 and Table S2 summarizes the corresponding parameters.

The phenol adsorption capacities of the four resins were similar at the three temperatures. The order of adsorption capacity was NDA-1800 > NDA-1801 ≈ NDA-1803 ≈ NDA-1802. The adsorption of micromolecules such as phenol is mainly achieved through π-πand dipole–dipole interactions. The resin adsorption capacity was greatly affected by the surface of the resin, and no ion exchange occurred. Compared with NDA-1800, the specific surface areas of the three amine-modified resins were decreased; thus, the adsorption capacities were lower. However, the specific surface area of the three amino-modified resins were similar, and their adsorption capacities were not significantly different.

The ABS acid adsorption capacity of the four resins was in the order of NDA-1801 ≈ NDA-1803 > NDA-1800 > NDA-1802 at all three temperatures. Compared with NDA-1800, the adsorption capacities of NDA-1801 and NDA-1803 were increased because of the increased polarity of the resins and the enhanced dipole-dipole and ion exchange interactions that occurred between the resin and the adsorbate, even though the specific surface areas were reduced after amination. The pore diameter distribution was slightly reduced, which was suitable for the adsorption of ABS acid molecules and increased the adsorption capacity. The adsorption capacity of the resin modified with secondary amines decreased slightly owing to the increased pore size distribution and the decreased specific surface area.

The RBB4 adsorption capacity of the four resins at all three temperatures was in the order of NDA-1802 > NDA-1803 ≈ ND-1801 > NDA-1800. Compared with NDA-1800, the adsorption capacities of the amine-modified resins markedly improved. The adsorption capacity of the secondary amine resin (NDA-1802) increased the most. This was because the presence of the polar functional group could be used for ion exchange and because the resin pore size remained large. The adsorption capacities of NDA-1801 and NDA-1803 increased owing to the presence of polar functional groups.

## 4. Conclusions

We have synthesized a series of mesoporous resins with different pore size distributions via suspension polymerization. These resins, which were suitable for the adsorption of macromolecules. The adsorption kinetics data were well-fitted by a pseudo-second-order rate equation, and the adsorption equilibrium data were described by a three-parameter multilayer adsorption model. The adsorption of small molecules reached equilibrium quicker than large molecules, and resins with smaller pores had a lower adsorption capacity for large molecules because of the pore blockage effect. NDA-1 and NDA-2 could efficiently adsorb phenol and ABS acid, while NDA-3 could efficiently adsorb RBB4. The resins were modified by amine groups. The presence of polar functional groups led to more efficient RBB4 adsorption, while the decreased specific surface areas led to decreased phenol adsorption capacities. Thus, a composite functional adsorbent resin with an improved ability to adsorb the macromolecule RBB4 was obtained. These mesoporous resins are promising candidates for application in removing organic contaminants from wastewater.

## Figures and Tables

**Table 1:** Characterization of resins.

Number	Specific Surface Area (m^2^·g^−1^)			S_ext_%	Pore Volume (cm^3^·g^−1^)			V_meso_%	Average Pore Diameter (nm)
	S_BET_	S_micro_	S_ext_		V_t_	V_micro_	V_meso_		
NDA-1	448.0	37.2	410.8	91.7	0.310	0.0098	0.3002	96.8	3.80
NDA-2	565.1	51.8	513.3	90.8	0.474	0.0148	0.4592	96.9	4.74
NDA-3	452.4	43.1	409.3	90.5	0.736	0.0213	0.7147	97.1	6.87
NDA-4	486.6	32.4	454.2	93.3	0.767	0.0091	0.7579	98.8	6.72

Note: S_BET_, S_micro_ and S_ext_ are the BET specific surface area, micropore area and non-micropore area, respectively. V_t_, V_micro_ and V_meso_ are the pore volume, micropore volume and meso-pore volume, respectively, when the relative pressure was 0.98. S_ext_% and V_meso_% are the percentage of the specific surface area of non-micropores and the percentage of the mesopore volume, respectively.

**Table 2:** Characterization of the modified resins.

Number	Specific Surface Area (m^2^·g^−1^)		Pore Volume (cm^3^·g^−1^)		Average Pore Diameter (nm)	Modification Methods	Polarity	Mean Particle Size (mm)	Residual Chlorine Content (%)	Total Exchange Capacity (mmol·g^−1^)
	S_BET_	S_micro_	V_t_	V_micro_						
NDA-1800	852.3	93.3	1.470	0.022	7.47	/	nonpolar	0.4–0.6	/	/
NDA-1801	630.6	84.1	0.944	0.021	5.99	Primary amine	polar	0.4–0.6	6.8	1.13
NDA-1802	628.4	54.7	1.230	0.009	7.86	Secondary amine	polar	0.4–0.6	7.1	1.49
NDA-1803	602.6	59.0	0.961	0.010	6.38	Tertiary amine	polar	0.4–0.6	5.4	5.40

**Table 3:** The corresponding parameters of the adsorption kinetic curves.

	Pseudo-First-Order Rate Equation			Pseudo-Second-Order Rate Equation		
	Q_e_ (L·mmol^−1^)	K_1_ (L·mmol^−1^)	R^2^	Q_e_ (L·mmol^−1^)	K_2_ (L·mmol^−1^)	R^2^
NDA-2 phenol	0.583	0.102	0.963	0.611	0.284	0.969
NDA-3 phenol	0.459	0.069	0.949	0.489	0.213	0.963
NDA-2 ABS acid	0.364	0.005	0.974	0.477	0.011	0.980
NDA-3 ABS acid	0.350	0.004	0.939	0.467	0.008	0.951
NDA-2 RBB4	0.267	0.002	0.957	0.384	0.003	0.958
NDA-3 RBB4	0.189	0.002	0.972	0.260	0.006	0.976

**Table 4:** The corresponding parameters of three-parameter multilayer adsorption model.

Resins	ABS Acid					RBB4		
	Q_m_ (mmol·g^−1^)	K_1_ (L·mmol^−1^)	K_2_ (L·mmol^−1^)	R^2^	Q_m_ (mmol·g^−1^)	K_1_ (L·mmol^−1^)	K_2_ (L·mmol^−1^)	R^2^
NDA-1	0.407	6.239	0.040	0.971	0.066	6.727	0.252	0.971
NDA-2	0.683	7.862	0.038	0.995	0.113	4.054	0.200	0.995
NDA-3	0.576	10.830	0.050	0.994	0.239	6.015	0.154	0.999
NDA-4	0.493	10.080	0.047	0.985	0.166	5.884	0.212	0.995

## Data Availability

Not applicable.

## Supplementary Materials

The following are available online, Figure S1: Characterization of the adsorbates, Table S1: The reagents used of synthetic resins, Figure S2: N2 adsorption isotherms of NDA-1 (a), NDA-2 (b), NDA-3 (c) and NDA-4 (d) resins at 77K (logarithmic scale (p/p0)), Figure S3: The infrared spectra of the four resins, Table S2: The corresponding parameters of three-parameter multilayer adsorption model.
