# Supplementary material for: Effect of Pore Size Distribution and Amination on Adsorption Capacities of Polymeric Adsorbents
Source: Molecules. 2021 Aug 30;26(17):5267. doi: 10.3390/molecules26175267 (PMC8433879; doi:10.3390/molecules26175267)
Supplement: Supplementary file 1 [file molecules-26-05267-s001.zip › molecules-1322565-supplementary.pdf]

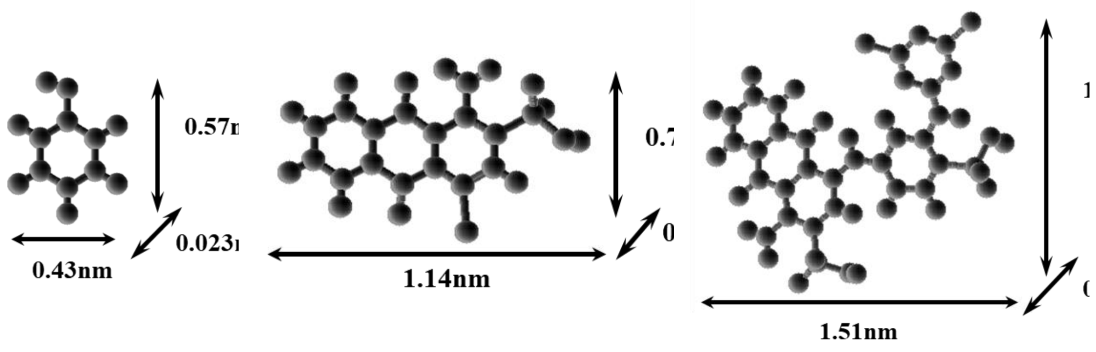

(**a**) Phenol [C_6H_6O] (MW: 94.1 g mol^{-1})

(**b**) ABS acid [$C_{14}H_8BrNO_5S$] (MW:382.2 g mol^{-1})

(**c**) RBB4 [$C_{23}H_{14}Cl_2N_6O_8S_2$] (MW:637.4 g mol^{-1})

Figure S1. Characterization of the adsorbates

Table S1. The reagents used of synthetic resins

Resins	Monomer concentration	Monomer: porogen	Composition ratio of porogen
NDA-1	83.5% DVB	1:1	Methylbenzene: hexadecanol=2:1
NDA-2	83.5% DVB	1:1	Methylbenzene: liquid-wax=2:1
NDA-3	50.0% DVB	1:1	Methylbenzene: liquid-wax=2:1
NDA-4	50.0% DVB	1:1	Methylbenzene: hexadecanol=2:1
NDA-1800	83.5% DVB	1:2	Methylbenzene: liquid-wax=9:1

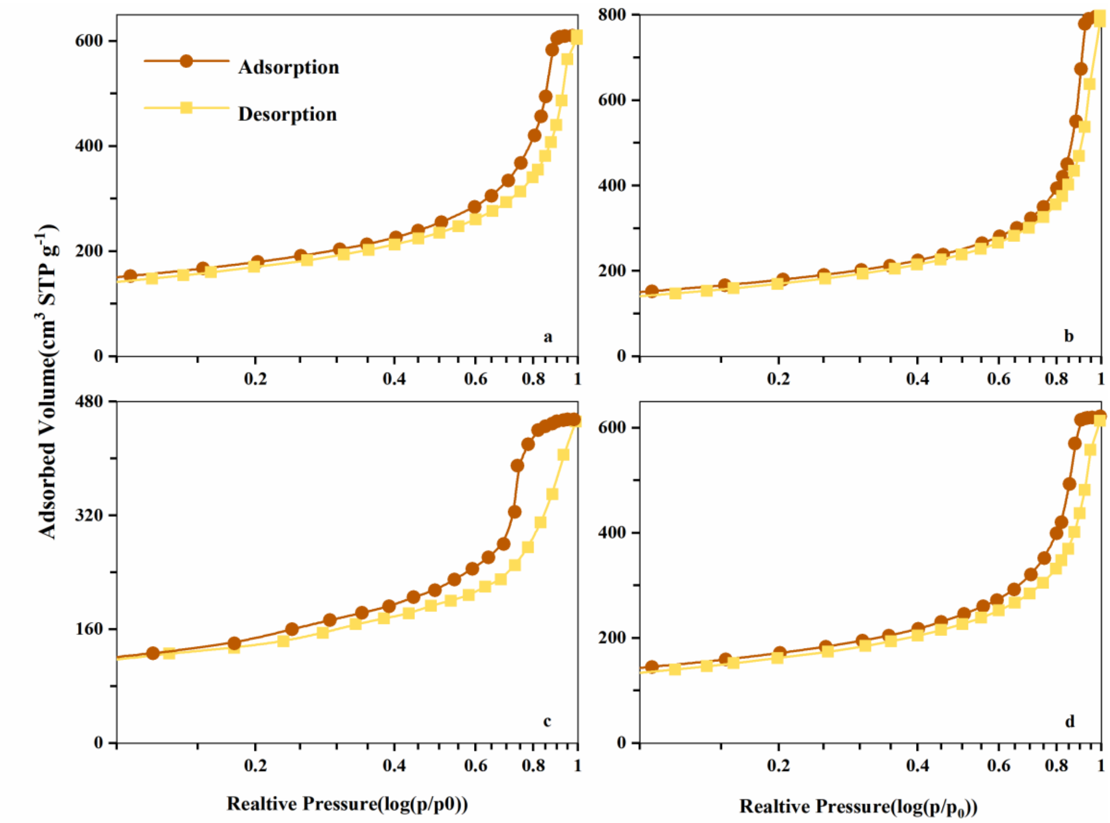

Figure S2. N_2 adsorption isotherms of NDA-1 (a), NDA-2 (b), NDA-3 (c) and NDA-4 (d) resins at 77K (logarithmic scale (p/p0))

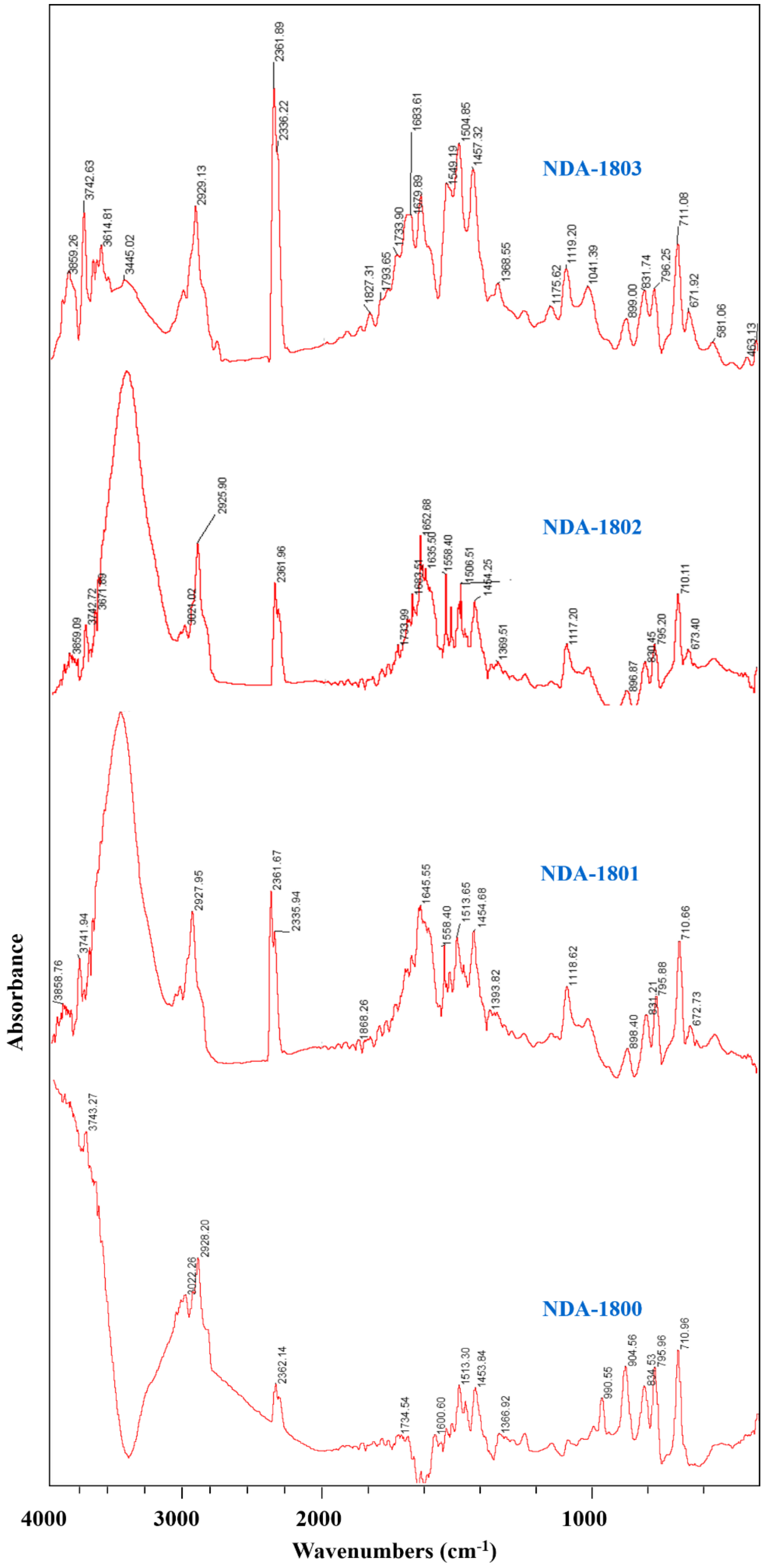

Figure S3. The infrared spectra of the four resins

Table S2. The corresponding parameters of three-parameter multilayer adsorption model

Temperature	Resins	Adsorbates	Q_m (mmol·g^{-1})	K_1 (L·mmol^{-1})	K_2 (L·mmol^{-1})	R^2
288 K	NDA-1800	phenol	31.990	0.009	0.003	0.981
		ABS acid	0.436	2.468	0.096	0.995
		RBB4	0.274	43.511	0.480	0.991
	NDA-1801	phenol	0.318	8.162	0.049	0.927
		ABS acid	0.415	365.888	0.123	0.975
		RBB4	0.384	52.466	0.509	0.984
	NDA-1802	phenol	0.425	0.772	0.040	0.992
		ABS acid	0.265	13.531	0.137	0.990
		RBB4	0.547	38.797	0.442	0.922
	NDA-1803	phenol	0.275	1.726	0.061	0.908
		ABS acid	0.404	990.766	0.115	0.982
		RBB4	0.522	10.407	0.286	0.874
303 K	NDA-1800	phenol	72.081	0.003	0.001	0.992
		ABS acid	0.340	2.287	0.161	0.998
		RBB4	0.256	37.635	0.484	0.995
	NDA-1801	phenol	0.310	1.234	0.040	0.968
		ABS acid	0.383	277.659	0.159	0.976
		RBB4	0.341	40.064	0.507	0.990
	NDA-1802	phenol	0.212	1.587	0.064	0.953
		ABS acid	0.227	10.970	0.202	0.963
		RBB4	0.532	24.333	0.388	0.910
	NDA-1803	phenol	0.278	0.672	0.047	0.901
		ABS acid	0.385	760.554	0.164	0.971
		RBB4	0.470	10.068	0.316	0.854
318 K	NDA-1800	phenol	30.147	0.004	0.001	0.991
		ABS acid	9.267	0.036	0.001	0.995
		RBB4	0.225	43.490	0.497	0.995
	NDA-1801	phenol	0.265	4.080	0.057	0.828
		ABS acid	0.361	82.485	0.143	0.963
		RBB4	0.306	22.129	0.466	0.985
	NDA-1802	phenol	0.229	2.147	0.057	0.800
		ABS acid	0.241	4.993	0.175	0.953
		RBB4	0.436	117.241	0.445	0.954
	NDA-1803	phenol	0.238	2.360	0.053	0.900
		ABS acid	0.335	335.688	0.170	0.975
		RBB4	0.299	341.179	0.468	0.992
